# First Encounters in Psychotherapy: Relationship-Building and the Pursuit of Institutional Goals

**DOI:** 10.3389/fpsyg.2020.585038

**Published:** 2020-12-15

**Authors:** Claudio Scarvaglieri

**Affiliations:** Department of Translation, Interpreting and Communication, Ghent University, Ghent, Belgium

**Keywords:** therapeutic relationship, process research, discourse analysis, client-centered therapy, psychodynamic therapy, alignment, conversation analysis, change research

## Abstract

This article examines how therapists and patients start building and managing relationships and pursue institutional goals at the same time. Based on a corpus of 6 audio-recorded therapies (client-centered therapy and psychodynamic therapy), I investigate first encounters between therapists and patients as the starting points of any therapeutical process and the place where a relationship between the interactants is established for the first time. Following a microlinguistic qualitative approach and applying methods from conversation analysis and discourse analysis, I show how therapists, on the one hand, try to align with patients to build a positive working alliance and, on the other hand, work to fulfill specific interactive tasks of therapeutic discourse which demand disaligning with the patients’ communicative activity and their interactive expectations. Specific interactive “jobs” that need to be fulfilled in psychotherapy are identified, namely the performance of institutional roles by the interactants, the establishment of an interaction structure and the pursuit of helpful change in the patient. I show at which places in the interaction therapists (dis-)align with the patients’ projected communicative activity and how aligning and disaligning are related to the interactive process and the establishment and performance of these interactive jobs. The analysis shows that, at the beginning of therapy, alignment and disalignment are both important processes for the following reasons: Aligning with the patient contributes to a positive relationship, which has been shown to be vital for successful psychotherapy, while disaligning introduces the patient to the specific discursive mechanisms that characterize therapeutic discourse and constitute the basis for its effectiveness. Overall, the paper argues that reducing therapy to a dichotomy between relationship and “technique” seems overly simplistic, as both aspects need to be handled and managed at the same time.

## Introduction

The therapeutic relationship between client and therapist is generally considered among the most important factors for successful psychotherapy and “the best and most reliable predictor of outcomes” (Ribeiro et al., 2013, 295). The importance of the therapeutic relationship is not restricted to any specific approach, and it has been shown to be “a reliable predictor of positive clinical outcome[s] independent of the variety of psychotherapy approaches and outcome measures” (Ardito and Rabellino, 2011, 1). The therapeutic relationship constitutes “the healing alliance between the client and the clinician” (Norcross and Lambert, 2018b, 303). In this paper, I investigate how the relationship between therapist and client can be operationalized and understood from an interactionist perspective, i.e., a perspective that is based on the documentation and analysis of the interaction between therapists and clients. I ask which aspects of the many interactional processes within therapy can be identified that considerably contribute to establishing and managing the therapeutic relationship and how therapists and clients affect and change their relationship at different points in the interactive process. I thus strive for both an interactionist understanding of the therapeutic relationship and an identification of vital interactional processes that impact the relationship, both negatively (threatening or weakening relationship and alliance) and positively (strengthening them).

In the following, I first describe the qualitative linguistic approach I follow in this paper. I then detail the linguistic methodology that I rely on as well as the data that form the basis of my analysis (section “Methodology and Data”). Based on a discussion of established approaches to the therapeutic relationship and interactionist-linguistic research on relationships in general, I suggest factors that allow us to identify crucial points in the establishment and management of the therapeutic relationship (section “Analytic Procedure”). In the section “Results,” transcripts from therapy sessions (psychodynamic therapy and client-centered therapy) are analyzed that show how therapists and clients establish and manage relationships. A number of practices are identified that are used – mainly by therapists – to uphold a positive therapeutic relationship while at the same time pursuing processes related to the institutional purpose of therapy. After discussing these findings and the theoretical and clinical implications in section “Discussion: Relationship Management and the Pursuit of Institutional Goals in Therapy,” I point out the limitations of this study and future directions (section “Limitations and Future Directions”).

## The Therapeutic Relationship From an Interactionist Perspective

A widely accepted definition of the therapeutic relationship is “the feelings and attitudes that the therapist and the client have toward one another, and the manner in which these are expressed” (Gelso and Carter, 1985, 159). Focusing on communicative (inter)action in this article, I add that the relationship is not only about “expressing” feelings and attitudes – as “expressing” suggests an explicit verbalization – but also about acting on them in some way, including non-verbally, for example with a head nod (Muntigl et al., 2013). A working therapeutic relationship in this sense does not necessarily include that therapist and patient like each other or feel sympathy for each other, but that they can work together in a therapeutically productive way.

Traditionally, the therapeutic relationship has been studied in great detail via questionnaires and checklists that are suitable to quantitative statistical analyses. While this methodology offers many advantages, including reproducibility, comparability and the attribution of exact impact scores to specific parts of the therapeutic process (Norcross and Lambert, 2018a), it has been pointed out (e.g., Muntigl and Horvath, 2014; Norcross and Lambert, 2018b; Muntigl, 2020; Storck et al., 2020) that it is difficult to address a number of relevant questions in this manner. First, questionnaires or checklists do not document the therapeutic relationship itself, but only what the participants are willing and able to (consciously) disclose about the interactive processes between them. Qualitative approaches, in contrast, document and investigate the process itself, which means that the “relationship in action” (Muntigl and Horvath, 2014, 327) becomes the object of analysis, including the contribution of the *patient* to the relationship. Whereas traditional measures are set up to treat therapy and the therapeutic relationship simply as the outcome of the therapist’s behavior and thus “neglect, relatively speaking, the productive contribution of the client to the therapy relationship” (Norcross and Lambert, 2018b, 307), an interactionist approach by design treats any interactive process, including establishing and upholding a relationship, as produced by all involved parties (Garfinkel, 1967; Sidnell and Stivers, 2013). Such an approach therefore allows us to study not only the patient’s impact on the therapeutic relationship, but also the techniques and methods deployed by the *therapist* to *manage* the patient’s contributions. Studying the therapeutic process as it unfolds also promises to further our understanding of *causal* connections within the therapeutic process. Whereas quantitative measures can reveal important *correlations* between different parts of therapy – like the therapist’s degree of empathy and its correlation with the success rate of therapy (Elliott et al., 2018) – detailed investigations of interaction in therapy have the potential to demonstrate how and why such factors contribute to helpful therapy. As a result, qualitative approaches allow for insights into how different aspects of the therapeutic relationship combine and work together – parts [like alliance vs. collaboration and self-disclosure vs. emotional expression (Norcross and Lambert, 2018a)] that quantitative research approaches often treat as separate, stand-alone practices (Norcross and Lambert, 2018b, 311), although in interactive reality neither therapist nor patient experiences or produces them separately. An interactionist approach therefore renders a picture of the therapeutic relationship that corresponds much more closely to the experience of the participants, while at the same allowing insights into the interactive processes that go beyond what participants themselves are able to consciously perceive while communicating.

## Methodology and Data

### Methods

In this paper, such insights into the details of the interactive process are sought based on established methods of linguistic conversation analysis (Sidnell and Stivers, 2013) and discourse analysis (Redder, 2008; Tannen et al., 2015). These approaches investigate communication as a joint process by all involved interactants who together produce certain activities (like telling a story or rendering an interpretation) or whole conversations and institutional processes (like an individual psychotherapy). As interaction is sequentially organized – i.e., in most cases a question will be followed by an answer, an invitation by an acceptance – specific patterns of interaction can be reconstructed through close interaction analysis (Redder, 2008). These approaches thus also make it possible to offer a detailed account of the “competences that ordinary members use and rely on in participating in intelligible, socially organized interaction” (Heritage and Atkinson, 1984, 1). Works within both approaches (see e.g., Ehlich, 1986; Heritage, 1998; Mondada, 2007; Redder, 2008; Stivers, 2008) have also shown that any or all features of an utterance or a turn may play a role in the doing of the action or constituting the activity. Therefore, interaction is documented and investigated in as much detail as possible, which includes documenting pauses, false starts, interjections (um, uh huh) and preface starters (well, okay). Another important characteristic is that these approaches base their analytic claims on what is publically available for viewing and hearing; that is, the original data on which the analysis is based are represented, which allows others to judge the validity of the analytic claims made. The methods applied here thus make it possible to offer a detailed description of the processes through which participants constitute and manage their relationships, and to reconstruct their activities and the institutional or individual goals they pursue through communication.

### Data

These methods are applied to a corpus of 6 audio-recorded short-term therapies (psychodynamic therapy and client-centered therapy) that were conducted in Germany. The patients were offered psychological treatment of up to 12 sessions after they had physically recovered from suicide attempts.

The corpus consists of six successfully completed therapies, three of which were carried out by a therapist trained in psychodynamic therapy (Delgado et al., 2015) and three by a therapist who uses the client-centered therapy approach (Rogers et al., 2013).

The conversations were transcribed following conversation analysis conventions (Jefferson, 2004), except that the lines are numbered like a musical score that depicts all participating “voices” (speakers) at a particular moment in the same line (cf. Rehbein et al., 2004). The horizontal position of the words thus indicates the order in which they were uttered. The transcription follows a medium level of abstraction and does not include all prosodic information, as the analysis focusses mainly on the verbal content of the exchanges.

### Ethics

The data presented here was collected by Norbert Dittmar. The study received ethics approval from FU Berlin and written informed consent was obtained from the participants for the publication of anonymized data. During and after data collection, clients had the right to cancel participation and opt out of the study. The recorded data were then deleted. Persons referred to in the transcripts, including clients and their family members, have been given pseudonyms.

## Analytic Procedure

As interactionist research on relationships shows (Goffman, 1959, 1966; Volosinov, 1930/1973; Locher and Watts, 2008; Linke and Schröter, 2017), any relationship is constantly monitored and managed by the participants. In principle, therefore, every communicative act has the potential to considerably modify or change a relationship. In a qualitative investigation of relationships, this might be taken to imply that everything that goes on between the participants has to be documented and analyzed in microlinguistic detail. As such an approach is not feasible in the context of this investigation, I instead suggest parts of the interactive process between therapist and patient that strongly contribute to the establishment and management of the therapeutic relationship. I thus identify ways to operationalize the therapeutic relationship within an interactionist research approach (cf. Muntigl and Horvath, 2014).

As any interpersonal relationship is initially established when people meet each other for the first time, I focus on *first encounters* in psychotherapy. This makes it possible to show how therapist and patient begin creating a relationship and how these crucial initial moments impact the interactive process between them.^1^ It also allows us to follow the development of their relationship and understand the interactive dynamics that underlie the changes and modifications it goes through.

To identify central relationship-building moments in these first encounters, I rely on a conceptual distinction first suggested by Hausendorf and Quasthoff (2005). They explain that, when communicating, participants perform specific interactive jobs, thereby creating a certain discourse type. For example, within the discourse type of storytelling, the interactants cooperatively pursue jobs such as demanding and giving attention to a longer turn by one of the participants, rendering a narrative turn and reacting during and after that turn (Sacks, 1995; Schiffrin, 2006; Norrick, 2010). If they consistently fail to complete these jobs – for example, if the aspiring storyteller is unable to get the attention of the recipients for a longer narrative turn – they will be unable to perform the overarching discourse type (in this case storytelling). Within institutionalized discourse types, we find that establishing and performing institutional roles (i.e., client vs. agent), establishing a certain institutional interaction structure (for therapy see e.g., Turner, 1972; Lakoff, 1982; Scarvaglieri, 2017; Peräkylä, 2019) and pursuing specific institutional goals constitute jobs that regularly need to be performed by the participants. In this paper, I specifically focus on *institutional roles* and the interactive processes in which they are established and subsequently performed, because these processes can have a specifically strong effect on the relationship between the interactants (Koerfer et al., 2018). Furthermore, they also affect other important parts of therapy, like the establishment of an interaction structure (i.e., performing certain roles also means establishing a certain interactional structure) and the pursuit of institutional goals (see below, sections “Results” and “Discussion: Relationship Management and the Pursuit of Institutional Goals in Therapy”).

While the concept of interactional roles is concerned with what could be called the meso-level of interaction – since the focus is mostly on *what* is done cooperatively, less on *how* this is done – the concept of *alignment* (Stivers, 2008; Lee and Tanaka, 2016) allows us to focus on the microlinguistic level of interaction. Alignment refers to the activity in progress and can be used to investigate whether the participants are taking part in the same kind of activity. Aligning responses join in the activity projected by an interactant and thus make cooperation possible “by facilitating the proposed activity or sequence, accepting the presuppositions and terms of the proposed action or activity, and matching the formal design preference of the turn” (Stivers et al., 2011, 21). While an aligning response thus takes part in the same activity, disalignment consists of no reaction at all or a reaction that pursues another activity. In ordinary conversation, this might be, for example, talking about the weather or the latest sports news instead of answering a question. Interactional alignment does not imply agreement: “… one can disagree but still cooperate with the general aims of the interaction” (Muntigl and Horvath, 2014, 329). For example, a negative response to a suggestion about a joint activity would constitute an act of disagreeing while aligning, whereas beginning to tell a story that is not related to the suggestion would be a form of disalignment, even if not explicitly disagreeing.

Concerning relationship, we may expect that participating in the same activity contributes to a stronger relationship, and that consistent interactional alignment by therapist and patient can thus serve as an indicator of a working relationship (Muntigl and Horvath, 2014). We may also expect that constant or repetitive disalignments in an interactional dyad will put a strain on the relationship. While these expectations serve as a starting ground, my analysis of specific instances of interaction in therapy demonstrates that disalignments are an important and essential part of the therapeutic process. The data show that an important aspect of relationship management by the therapist is dealing with what could be deemed “necessary” disalignments and being able to uphold a working therapeutic relationship with the patient.

In the following, I investigate extracts from first encounters in therapy (macro-perspective), as that is where every relationship is initially formed and because early relationship formation has been shown to be particularly important for successful therapy (Horvath and Bedi, 2002, 55). Within these encounters, I specifically focus on the establishment of different institutional roles by the participants (meso-perspective) and ask whether participants align interactively and cooperate in the same activity or disalign at certain places in the interaction (micro-perspective).

## Results

### Patients as Experts

In verbally oriented therapies – such as client-centered therapy, psychodynamic therapy and psychoanalysis – patients and therapists perform different roles (Scarvaglieri, 2017). An important aspect of these roles concerns epistemic authority – that is, the idea that interactants have different types of knowledge about different domains of reality as well as different degrees of certainty about these knowledge domains, and that they will index domains about which they have specific knowledge in conversation (Labov and Fanshel, 1977; Heritage and Raymond, 2005; Heritage, 2013). In most cases, for instance, interactants will claim epistemic authority on their own mental processes, thereby marking “territories of self” (Heritage, 2013, 382). Failing to constantly maintain “such territories is to risk deracination and, at the limit, even depersonalization” (ibid.). In verbally oriented psychotherapy, the client needs to claim epistemic priority about their specific biographical experience, and thus to perform the role of expert in regard to their own emotions, perceptions and specific events they were part of. In general, therefore, the client’s role conforms to conventional principles of epistemic authority, as participants are generally expected to know best about their own experience. The therapist, in contrast, will often take on the role of someone who is able to contextualize the patient’s experiences and offer explanations for or a different understanding of them (cf. Muntigl et al., 2013; Weiste et al., 2016; Scarvaglieri, 2019a). Performing this role can thus present a challenge to established principles of epistemic authority, according to which the patient, as the person who had these experiences, is best suited to understanding and explaining them. These roles thus present challenges for both participants and can potentially strain their relationship. Nevertheless, the following extracts show that these roles are established and performed immediately at the beginning of a therapy.

**FIGURE 1:**
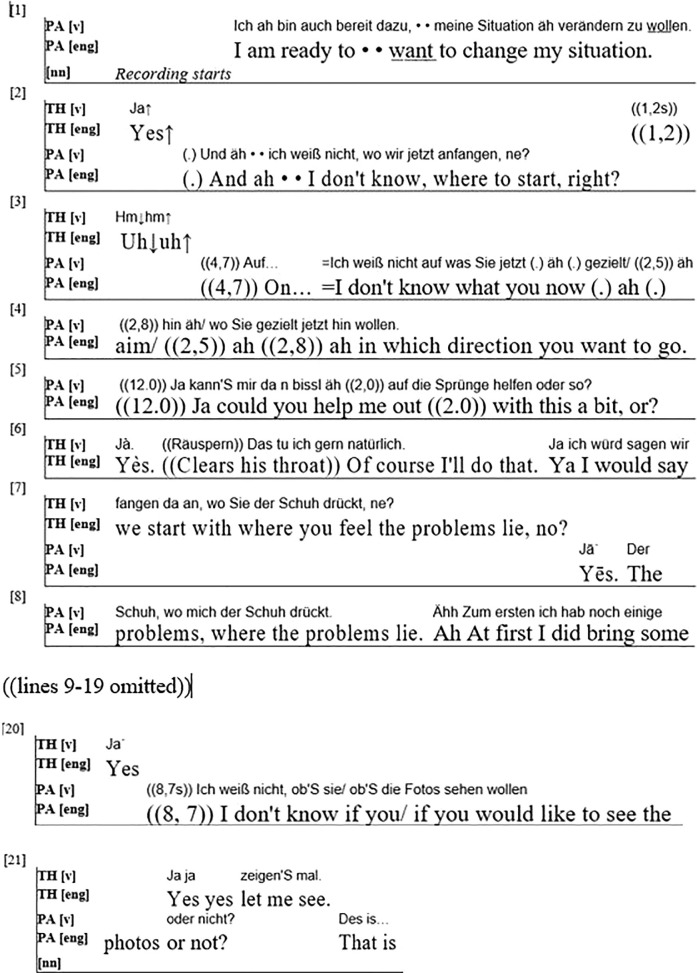
(PA = Patient; TH = Therapist).

The first extract (Figure 1) is taken from the first session of one of the client-centered therapies. The patient is a middle-aged man who shows symptoms of alcohol use disorder during the therapy.^2^ I present the very first utterances that were recorded of this therapy (greetings or welcomings were not recorded).

The patient first states his willingness to change. He then makes three attempts to get the therapist to start off the conversation by choosing the topic they should talk about. At first he mentions that he himself does not know what to talk about or “where to start” and adds the question tag “right” (German “ne”) to draw a response from the therapist (line 2). The therapist reacts after a brief pause with an acknowledgement token (“Uh uh” (line3)) but does not accept the turn. After another pause, the patient again mentions a lack of knowledge, this time about “the direction” the therapist might “want to go” (line 4). The therapist again does not react verbally, which leads to a long pause and the patient now explicitly asking whether the therapist could “help me out ((2.0)) with this a bit” (line 5). The therapist reacts to this question by asking the patient to “start with where you feel the problems lie”^3^ (line 6–7). Thus, the therapist again refuses to choose a topic, instead giving this task back to the patient. The patient demonstrates difficulty with this reaction as he repeats the therapist’s utterance, still searching for a suitable starting point.

We thus see that, from the start of the first session, the therapist treats the patient as the expert on his own experience and perception and as the only person able to choose the relevant topics in the session. He does this implicitly, by not reacting to the patient’s invitations to choose a topic, and explicitly by asking the patient to choose a topic himself and, a bit later in this session, explaining to him his version of Freud’s “fundamental rule” (Freud, 1900/1913). In this short sequence alone, the therapist thus uses multiple interactional devices – non-reaction, evasive responses and explicit explanations – to establish the patient’s role as the expert on his own life experience.

On the micro-level of linguistic investigation, we find that disaligning with the patient’s projected activity plays a big part in this initiation to a new discourse structure and the institutional distribution of roles. The patient repeatedly tries to project a common activity in which the therapist tells the patient “where to start” and “in which direction to go,”^4^ but the therapist twice reacts only minimally through acknowledgement tokens or not at all, he does not cooperate in the activity. This changes only when the patient poses an explicit question asking for “help.” Now the therapist answers and thus formally aligns with him, although in his response he suggests pursuing another activity than the one proposed by the patient (the patient choosing the topic instead of the therapist suggesting one). We thus find that the therapist first disaligns, and that when he re-aligns (cf. Muntigl and Horvath, 2014, 340–41) formally – by responding to the patient’s questions – in his answer he shows that he still disagrees with the patient’s suggestion about their joint activity.

The patient at first seems to be irritated by this behavior, which is indicated by the long pauses he makes within and between utterances (lines 3, 4, 5) and by false starts (line 3) and self-repairs (lines 3, 8). A bit later though, the patient starts adjusting to the new role, by asking the therapist whether he wants to see the photos the patient brought to the session (lines 20–21). In everyday interaction with friends or colleagues, mentioning that one brought photos along would commonly lead to the other side suggesting to share them and discuss them together. After mentioning the photos, the patient here does not wait for such a reaction by the therapist. Instead, he asks himself whether the therapist wants to see them: “I don’t know if you/if you would like to see the photos or not?” (lines 20–21). Although he again uses a “I don’t know” construction (cf. line 3, 4), this time it is rendered as a “yes/no” question that is supplemented by a question tag (“or not”), which makes it clear that a response is expected. This time, the therapist reacts quickly and agrees to look at the pictures (“Yes yes. Let me see.” (line 21)), even before the patient finishes his turn. The patient thus appears to have made a first step in adjusting to his new role and in understanding that the therapist will not always follow established patterns of everyday interaction. Therefore, by explicitly asking for it, the patient himself ensures that things are discussed that are important to him.

Overall, in these few utterances from the beginning of the therapy we see a therapist immediately establishing institutionally differentiated roles and using a number of interactional and linguistic devices to do so, including disaligning, re-aligning and disagreeing. While it is difficult to discuss the intentions of the therapist or the reasons for his behavior based on conversational data, the effects of his behavior consist in introducing a specific conversational structure and the different roles of therapist and client. The patient’s reactions suggest that he is slowly adapting to these changes.

**FIGURE 2:**
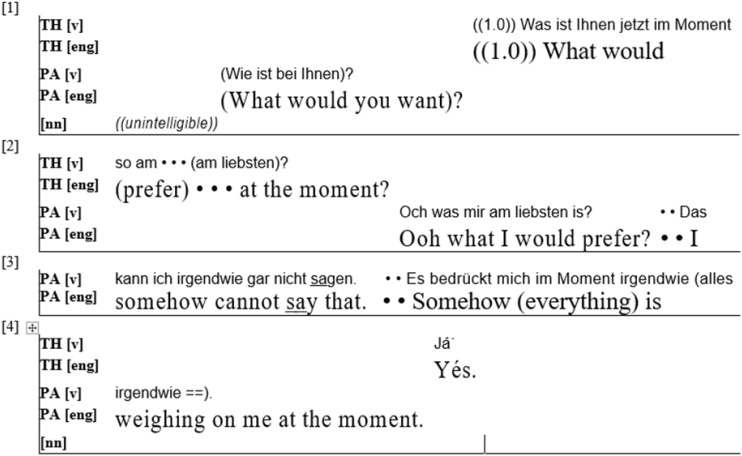
(PA = Patient; TH = Therapist).

The second extract (Figure 2) shows similar behavior, and it also demonstrates the difficulties patients have in adjusting to their role. The extract stems from the first session of a psychodynamic therapy with a female patient in her twenties. I again present the very first utterances of the therapy.

After a short, unintelligible sequence, the patient asks what the therapist wants to talk about.^5^ As in the first example, the therapist, after pausing briefly, does not react by suggesting a topic. He disaligns with the patient and instead gives the question back to the patient and asks her what she “would (prefer) at the moment” (line 1–2). In another striking parallel to the first extract, the patient then repeats the therapist’s question, thereby giving herself time to think. She then states that she cannot decide, and that “somehow (everything) is weighing on me at the moment” (lines 3–4). This example thus again shows a therapist disaligning with the patient and refusing to choose a topic, instead insisting that the patient choose for herself. In both examples, the therapists immediately work to establish a certain role distribution. Patients are made to choose a topic, demonstrating to them that they are the experts on their own experience and best able to decide what is important in therapy. To establish this role, therapists disalign with the patient and thus accept a potential strain on the relationship.

### Therapists as Experts

While therapists work on making patients talk about their own experiences, they sometimes present suggestions about the context of the patient’s experience or possible explanations for it (Peräkylä, 2004, 2005, 2019). Therapists thus work to establish themselves in a role where they serve as experts in understanding the events and experiences that the patient has just described.

**FIGURE 3:**
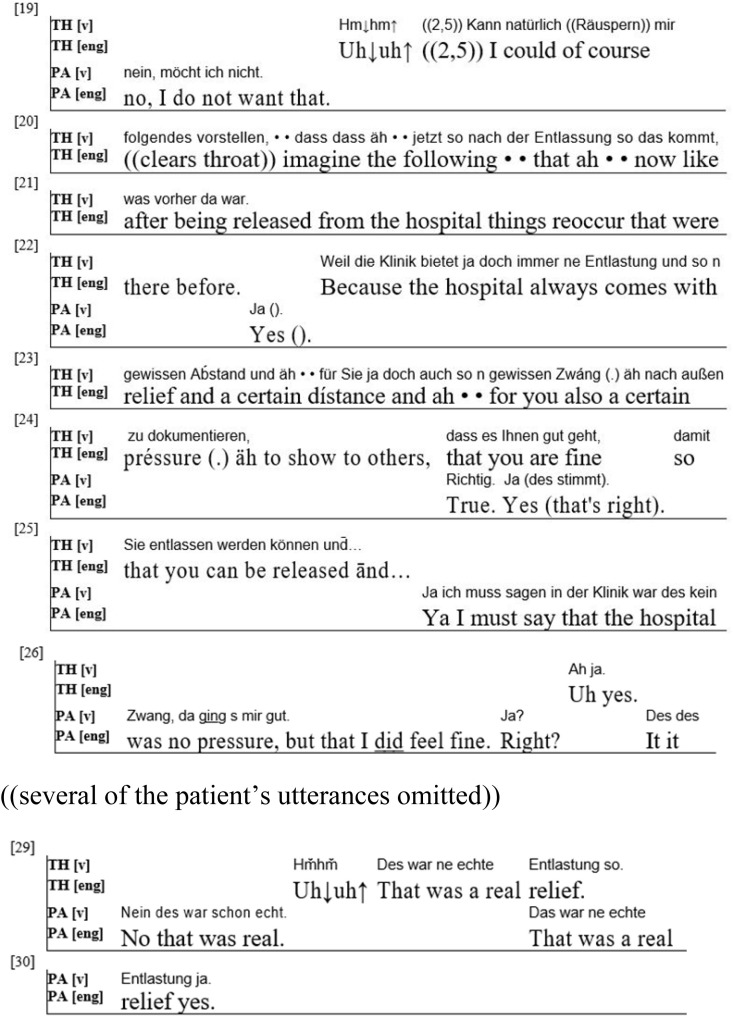
(PA = Patient; TH = Therapist).

The third extract (Figure 3) stems from the same session of the same (psychodynamic) therapy as the second. The patient had mentioned that, after physically recovering from her suicide attempt and being released from the hospital, she began to feel much worse than during her recovery in the hospital and again experienced suicidal thoughts. The therapist then offers an explanation for why these thoughts might have reoccurred.

The patient finishes her turn by stating that she wants to get off of her medication (“I do not want that” (line 19) refers to the medication). As she does not take the turn after a pause of 2.5 seconds, the therapist assumes the right to talk and offers a possible explanation for her negative feelings after returning home from the hospital. He “imagine[s]” that at home “things reoccur that were there before” (lines 20–21) – that is, that the patient is again in the pathogenic biographical situation in which her problems appeared in the first place. He also suggests that the hospital had certain advantages, like “relief,” “distance” and “a certain pressure” (lines 23–24), that were lost after the patient left the hospital.

With this, the first explanatory turn of the therapy, the therapist starts to establish himself as someone who is willing to contextualize the patient’s experience and offer potential explanations. He does this even though the patient is projecting another activity: she had mentioned her negative feelings and then moved on, focusing instead on her goals and expectations for the therapy. By changing the activity and the topic of conversation, the therapist here disaligns with the patient. He talks about the reasons for her emotions instead of her goals in therapy. We then see that the patient accepts this change and aligns with the new activity. She thus accepts the role the therapist takes on. She offers a number of agreement tokens [“Yes” (line 22) “True,” “Yes that’s right” (both line 24)], but later denies that she felt pressure while in the hospital, instead stating that she felt safe and relaxed there. She thus aligns with the new activity but disagrees with the therapist’s assumption about her feelings. She thereby claims epistemic authority (Heritage, 2013) for her own experiences and feelings. The therapist, by rendering a suggestion about the roots of her feelings and her experience in the hospital, had turned the patient’s epistemics into a domain over which he also claimed authority, but the patient, by rejecting parts of the therapist’s claim, restores a hierarchy according to which she preserves epistemic priority. After such a (partial) rejection of an explanatory utterance (Peräkylä, 2004), the therapist has various options, including elaborating on his explanation (Peräkylä, 2005), insisting or retreating (Muntigl et al., 2013). In this case, the therapist retreats [he accepts her rejection (lines 26, 29)], thus demonstrating to the patient that he accepts her epistemic authority about the content of her feelings. Nevertheless, the therapist presented himself here as someone who is able to offer explanations for the patient’s inner processes. The patient accepted and aligned with this activity, and she also accepted the therapist’s proposed explanation, but rejected the part where he tries to describe – not explain or contextualize – her feelings while being in the hospital. We thus find that both interactants here cooperatively establish themselves and each other as experts for different parts of the patient’s experience: the patient as the expert regarding the experience itself (the “what” of her feelings), the therapist as the expert regarding contextualizing and understanding this experience (the “why” of the feelings). This example thus shows how both participants work to establish separate domains of authority concerning the patient’s epistemics. We also notice that this establishing of roles includes processes of disalignment and realignment with the patient’s activity by the therapist.

**FIGURE 4:**
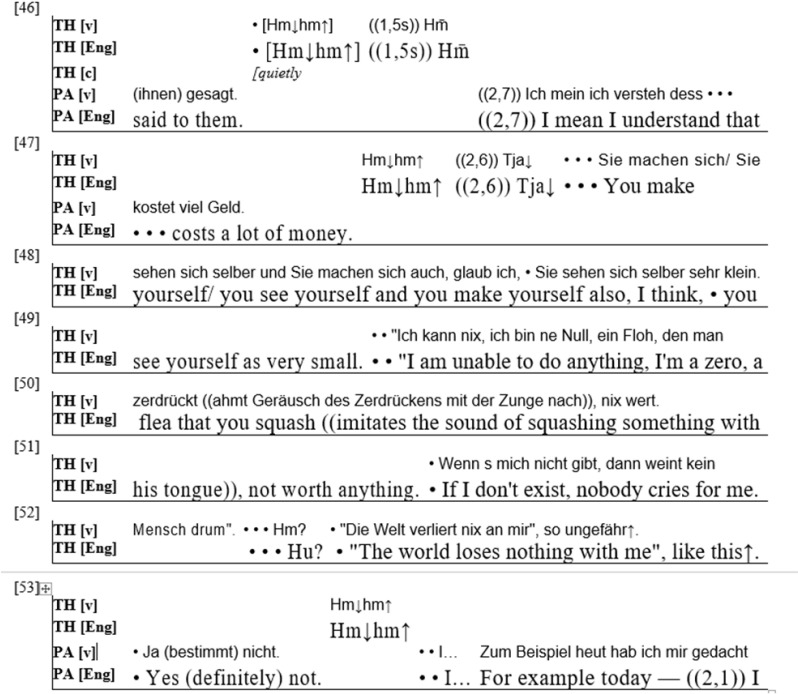
(PA = Patient; TH = Therapist).

To illustrate these processes further, I point to an example from a third therapy, the first session of another client-centered therapy (Figure 4). Before the extract presented here, the therapist and the patient had examined the patients emotions at home vs. during her stay in the hospital (this extract does thus not start at the beginning of the session). The patient is a woman in her fifties who has a very difficult relationship with her husband and shows signs of a depressive disorder.

The session had started with the patient expressing how well she had felt while in the hospital, which, as in the third example, contrasts strongly with her feelings after returning home. Together, the therapist and the patient then examine the reasons for the positive emotions in the hospital, with the therapist repeatedly asking the patient to search for explanations and also offering potential reasons himself. Then the patient describes that she left the hospital against her will. She says she had mentioned that she had wanted to stay longer (line 45, not represented), but had to leave anyhow. In lines 46 and 47, she expresses understanding for this, as a hospital stay “costs a lot of money.” Then the therapist comes in and changes the activity that the two interactants are pursuing. Whereas before the patient was mainly describing her feelings as well as specific events (like talking to the doctors, leaving the hospital), the topic shifts to a general description of the patient’s character traits. The therapist sketches a picture of the patient’s view of herself (“you see yourself,” line 48) and of her behavior toward herself (“you make yourself,” ibid). After this general characterization, according to which the patient sees herself as and makes herself “very small” (line 49), the therapist uses auditory depictions (Scarvaglieri, 2013b; Clark, 2016) and thinks aloud from the patient’s perspective (Yamaguchi, 2005; Tay, 2013, 112) to sketch the picture of a person who has very little self-regard. He closes by stating from the patient’s perspective that the world would lose nothing if she ceased to exist, to which the patient agrees empathically.

We thus find the therapist changing the activity that the interactants had pursued together for a while. His disalignment here allows him to present a characterization of the patient and “reflect” to her, in a detailed and quite compact manner, his impressions of her. This activity is part of the client-centered technique and aims to make patients aware of parts of their self that they are as of yet unaware of. It is supposed to contribute to an image that patients have of themselves that more closely resembles the actual structures of their self. According to the underlying therapy theory (Eckert et al., 2006; Rogers et al., 2013), a more “realistic” view of the self will significantly contribute to processes of healing. The therapist’s disaligning here is thus related to what, in traditional terms, could be called “technique” – it allows the therapist to present his impression of the patient. In doing so, the therapist accepts the risk this might pose to the therapeutic relationship with the patient, and he introduces himself as someone who is able to describe the patient in significant detail and in quite unambiguous terms. The example thus shows that therapists manage relationships and “technique” – presenting different and new knowledge to the patient and establishing themselves as experts in explaining and understanding the patient’s experience – at the same time, in the same utterances, and that both aspects are relevant to understand the therapeutic process.

### Managing Resistance

**FIGURE 5:**
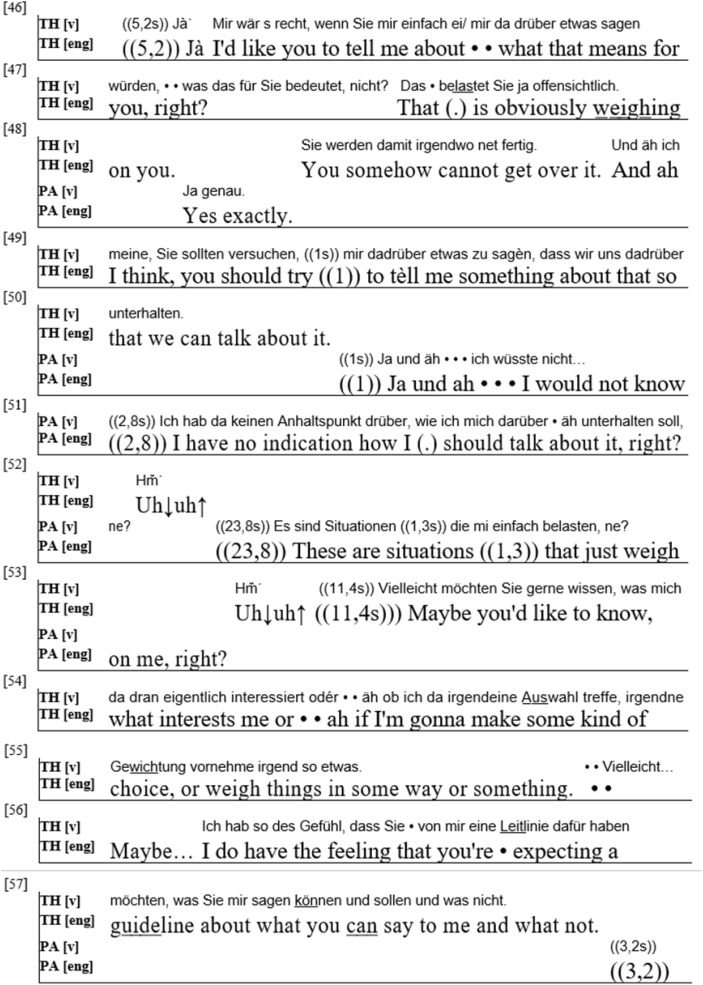
(PA = Patient; TH = Therapist).

In the following, I present an example in which the patient shows himself unable (or unwilling) to perform the role of the expert on his biographical experience. This example (Figure 5) stems from the same therapy (first session) as example 1 (middle aged male patient, alcohol abuse).

The excerpt starts with the therapist pointing out the relevance of events that the patient had just talked about (the suicide of his sister, the divorce from his wife). He mentions that the patient seems unable to “get over” these experiences (line 48) and asks him to elaborate on them. The patient replies that he does not know “how to talk” about this and that these “situations” “just weigh” on him (lines 51–52). He leaves a very long break between his utterances and does not expand on them. The therapist initially reacts with supportive continuers and by leaving the floor to the patient – overall, almost 40 seconds pass in which nothing happens besides the patient expressing an inability to talk. By refusing to accept the turn, the therapist thus insists on the therapist/client role distribution. As the silence stretches, however, the therapist does assume the turn. After initially refusing to align with the patient’s projected activity (the therapist helping the patient to talk about his experience), he formally re-aligns with the patient, simply by accepting the right to speak. However, he does not offer any suggestions about how the patient might feel regarding the life experiences that had been mentioned, he thus does not do “the patient’s job.” Instead, he talks about what the patient might want or expect from the therapist in the current situation (“I do have the feeling that you’re expecting a guideline about what you can say to me and what not” (55–57)). The therapist thus meets the patient halfway – he formally re-aligns by taking the turn and temporarily freeing the patient of the pressure to speak, but he does not offer suggestions about the patient’s feelings regarding the experiences in question.

This example shows a therapist reacting to a patient who does not perform his role. After at first disaligning with the patient’s projected activity, he later re-aligns formally by accepting the turn, but changes the topic of the conversation. The therapist thus on the one side alleviates the pressure on the patient to speak about something he clearly has problems addressing, and on the other side keeps the focus on the patient’s inner experience. We see how the therapist here tries to find a middle ground between making the patient speak about himself and establishing institutional roles and structure, and the need to uphold a working relationship, which includes adapting to the patient’s needs and (in)abilities in the therapeutic situation.

Overall, this example adds to our finding that therapists pursue aspects of relationship and technique at the same time and that they adapt their behavior according to the patient’s actions, while still striving to achieve institutional und interactional goals.

## Discussion: Relationship Management and the Pursuit of Institutional Goals in Therapy

In the five extracts just examined, we have found that therapists frequently disalign with the patient’s projected activity, and that they also perform actions that could be seen as infringing on the patient’s epistemic authority. Both of those practices – repeatedly disaligning and contesting epistemic authority – can be problematic for a functioning therapeutic relationship (Muntigl et al., 2013; Muntigl and Horvath, 2014; Weiste et al., 2016). This seems even more relevant at the beginning of a therapeutic process, as there is no interactional history between the two participants, no shared common ground to rely on. What is more, the therapists here are dealing with persons who are recovering from suicide attempts and have a special need for a strong and reliable relationship with a caregiver. The therapist’s behavior may thus seem counterproductive. This impression changes, however, when we focus on the accompanying actions that therapists perform in these situations, and when we try to understand which objectives the therapists are pursuing when they disalign and challenge epistemic authority.

First, we see that the therapists modify and adapt their disaligning and challenging utterances, in the third example by framing them not as fixed knowledge about reality, but as possible imaginations (“I could imagine that”; cf. Muntigl and Horvath, 2014, 331), and by downgrading the propositional content (“a certain distance,” “a certain pressure”; concerning the framing of potentially controversial content cf. Vehviläinen, 2003, 578). Other means that have a similar effect are expansions that widen the topic of the talk and thus make it easier for the patient to identify elements with which they can agree (Peräkylä, 2005, 173–74), the explicit presentation of new knowledge as suggestions, proposals or in the form of a question (Vehviläinen, 2003, 597; Muntigl et al., 2012, 126; Scarvaglieri, 2013a, 159–62), various forms of hedges (such as “epistemic downgraders” (Muntigl and Horvath, 2014, 332; see also Ehlich, 1990; Weingarten, 1990; Weiste et al., 2016) and try-markers (Sacks and Schegloff, 1979, 18).

These linguistic devices either “weaken the illocutionary force” (Ehlich, 1990, 219; my translation) of an utterance, for example by turning an assertion into a question or a statement into an assumption, or reduce the scope of the propositional content of these disaligning and potentially controversial statements, which leads to less far-reaching statements that in principle should be easier for the patient to accept (Scarvaglieri, 2013a). Both of these techniques – weakening the illocutionary force and lowering the propositional weight of an utterance – can potentially further a critical examination of the utterance’s content by the patient and communicate to the patient that they decide whether to accept it or not. With these techniques, therapists strive to mitigate the potentially problematic relational consequences of disaligning utterances or utterances that infringe on epistemic authority (cf. Weiste et al., 2016).

In addition to modifying disaligning and challenging utterances, therapists also use interactional means – i.e., specific actions – to mitigate the relational effects of any problematic turn. They attempt to realign with patients at a later point in the interaction (extract 3), explicitly retreat from statements that challenge epistemic authority (extract 3) (Muntigl et al., 2013) and explain disalignments (extract 1) to mitigate their effects on the therapeutic relationship. There are thus linguistic and interactional devices that make it possible for therapists to verbalize new and different knowledge and for patients to discover new insights, and to at the same time uphold a working relationship with the patients.

Second, in the examples above, disaligning actions by the therapists are related to the introduction of specific roles and an institutional structure of interaction. In the most basic terms, therapy consists of two processes (Scarvaglieri, 2017): the patient verbalizes their biographical knowledge, and then therapist and patient discuss and thereby change or adapt this knowledge. We have seen that the therapists’ disaligning actions serve to introduce and support this structure and the respective roles of patient and therapist. The therapists did not respond to patients’ questions about where to start the therapy in a conventional manner – i.e., by suggesting a topic – and instead gave the question back to the patient, kept silent or explained in detail that such questions would in principle not be answered. Disalignments are thus related to establishing the client as the expert regarding their personal experience who knows best what is important to them and what thus needs to be discussed in therapy. Other examples of disalignment observed above include therapists commenting on the experience just described by the patients, thereby establishing themselves as persons able to contextualize the patient’s experience and introducing the “second” part of therapy, the discussion of the patient’s experience. These disalignments are thus also related to establishing the characteristic discursive structure of therapy and its specific distribution of roles (therapists as experts in contextualizing and understanding experience, patients as experts in choosing from their biographical experience). Disalignments thus help create the specific interactive character of therapy.

Third, by disaligning with the patient’s projected activity, therapists are able to introduce new knowledge and a diverging perspective on the patient’s experience. This can be seen as an essential element of verbally oriented psychotherapy (Vehviläinen, 2003; Peräkylä, 2004; Weiste et al., 2016) that allows patients to change their perspective of self and others (Scarvaglieri, 2019b) and to understand their biographical situation differently and in terms that support a productive handling of their situation (Scarvaglieri, 2019a). In this regard, disalignments are thus related to the institutional purpose of therapy, i.e., to promote positive mental and behavioral change in the patient (Graf et al., 2019; Pawelczyk, 2019).

The data not only showed that therapists use several means to reduce the effect of disalignments on the therapeutic relationship, but also that patients are able to adapt to these changes, as early as in the first session. As patients adjust and become used to their role and the specific conversational structure, they will expect disalignments by the therapists and thus will not perceive them as particularly problematic. The potentially negative impact of any disalignment on the relationship between therapist and client therefore decreases as the therapy proceeds and patients get used to its interactional structure. The disalignments early in the therapy, as discussed here, thus establish a basis for therapeutic work in the later sessions.

Overall, I have found that alignments and disalignments by therapists are related to not only relationship management, but also the characteristic structure of therapeutic discourse (roles, interactional structure) and its purpose (achieving mental and behavioral change through communication). The data show that therapists focus not only on establishing a working therapeutic relationship with patients – not even in the initial moments of the first session, not even with psychologically unstable patients – but also, and equally, on pursuing institutional goals and establishing interactive roles and a specific structure of interaction. These objectives demand interactive disalignments between therapist and patient that have the potential to harm their relationship. Therapists pursue them nevertheless, which demonstrates the value they place on them. To mitigate the effects of disalignments and reconcile the pursuit of institutional goals with relationship management, therapists use specific interactional and linguistic devices that are designed to activate patients and show them that they can accept or reject such utterances. Therapy thus shows itself as a complex balancing act in which processes of relationship management, institutional goals and institutional structure need to be pursued at the same time. Each action, whether it is part of a therapeutic “technique” or not, affects and regulates the therapeutic relationship, and the therapists’ actions take this into account. The data thus show that therapy as both a clinical process and an object of scientific study cannot be reduced to a dichotomy between relationship and technique, and that both aspects have to be considered at the same time, as they both constitute the basis of any therapy and are regularly pursued and managed at the same time.

## Limitations and Future Directions

While the present study has shown how therapists and clients co-manage the relationship and at the same time pursue institutional goals, it has illustrated its findings through a limited number of examples. Although these examples are backed by analyses of a broader corpus that consists of six different therapies, the research presented here has to be understood as exploratory in nature. Taking data from other backgrounds (including other types of therapy) into account might well lead to observations of further and different ways of introducing and managing the therapeutical relationship.

When operationalizing the therapeutic relationship, decisions were made to focus on specific aspects. While these decisions are firmly grounded in interactionist relationship research, we can expect to find further important practices of relationship management when focusing on different aspects. Along the lines of the above argument about the convergence of “technique” and relationship (from a theoretical and empirical perspective), it would seem promising to focus, for example, on interpretations and similar therapeutic interventions and the relational challenges these activities present and how these challenges are dealt with.

A further limitation concerns the fact that, due to the limited scope of this study, it was not possible to examine how different relationship-management techniques change through the course of a therapy or how the initial establishing of the relationship influences its development over time. Such a longitudinal, supra-session (cf. Bercelli et al., 2013; Voutilainen et al., 2018) investigation of relationship management would constitute another fruitful avenue in the study of the therapeutic relationship.

Finally, to overcome the general limitations of any qualitative study while simultaneously preserving its ability to make the relationship itself visible, it would seem promising to combine qualitative research with quantitative measures. It would for example seem worthwhile to code different types of disalignments by the therapist (e.g., whether they concern the patient’s role or the therapist’s role, whether they occur after a pause in the conversation or during or immediately after the patient’s turn) and the types of reactions these provoke (e.g., acceptance, resistance, silence) to further understand the distribution of disalignments and their effects on interaction (cf. Ribeiro et al., 2013). This would allow us to study the interactional impact of the discussed measures on a broader scale and lend further evidence to the emerging picture of therapy as a complex combination of individuals and institution, processes and outcome, and relationships and technique.

## Data Availability Statement

All datasets generated for this study are included in the article.

## Ethics Statement

The studies involving human participants were reviewed and approved by the ethics committee of FU Berlin. The participants provided their written informed consent to participate in the study. Furthermore, persons referred to in the transcripts, including clients and their family members, have been given pseudonyms.

## Author Contributions

CS performed the analysis of the data and wrote the whole article.

## Conflict of Interest

The authors declare that the research was conducted in the absence of any commercial or financial relationships that could be construed as a potential conflict of interest.

## Footnotes

1 Even the first session of psychotherapy, though, does not start with a blank slate, as patients and therapists hold expectations that can affect interaction and relationships. In the cases discussed here, this becomes visible when patients bring along photos or letters to show to the therapist (Figure 1) or when they utter their expectations about the objectives of the therapy early in the first session (Figure 3). While these instances show that patients think about therapy before the first session and prepare for it, the relational expectations that they can be expected to bring along as well are more difficult to investigate based on conversational data. I therefore refrain from discussing these in detail in this paper.

2 The alcohol problems become manifest as the therapy continues. In the session documented here, the patient was not drunk or noticeably under the influence of alcohol.

3 In German, this is formulated as a phrase that literally means “where the shoe squeezes you.”

4 From the perspective of metaphor analysis (Buchholz, 1998; Buchholz et al., 2008; Tay, 2013) it is noticeable that the patient here repeatedly frames therapy as process of joint movement that has a starting point and a direction.

5 Parentheses indicate that the transcriber was not fully sure about the content of the utterance.

## References

[B1] Ardito R. Rabellino D. (2011). Therapeutic alliance and outcome of psychotherapy: historical excursus, measurements, and prospects for research. *Front. Psychol.* 2:270. 10.3389/fpsyg.2011.00270 22028698 PMC3198542

[B2] Bercelli F. Rossano F. Viaro M. (2013). Supra-session courses of action in psychotherapy. *J. Pragmat.* 57 118–137. 10.1016/j.pragma.2013.08.001

[B3] Buchholz M. (1998). Die metapher im psychoanalytischen dialog. *Psyche* 52 545–571.

[B4] Buchholz M. Lamott F. Mörtl K. (2008). *Tat-Sachen: Narrative von Sexualstraftätern.* Gießen: Psychosozial-Verlag.

[B5] Clark H. H. (2016). Depicting as a method of communication. *Psychol. Rev.* 123 324–347. 10.1037/rev0000026 26855255

[B6] Delgado S. Strawn J. R. Pedapati E. V. (eds) (2015). *Contemporary Psychodynamic Psychotherapy for Children and Adolescents: Integrating Intersubjectivity and Neuroscience.* Heidelberg: Springer.

[B7] Eckert J. Biermann-Ratjen E.-M. Höger D. eds (2006). *Gesprächspsychotherapie: Lehrbuch für die Praxis.* Heidelberg: Springer.

[B8] Ehlich K. (1986). *Interjektionen.* Tübingen: Niemeyer.

[B9] Ehlich K. (1990). “Zur Struktur der psychoanalytischen Deutung,” in *Medizinische und Therapeutische Kommunikation: Diskursanalytische Untersuchungen*, eds Ehlich K. Koerfer A. Redder A. Weingarten R. (Opladen: Westdeutscher Verlag), 210–227. 10.1007/978-3-663-14675-9_15

[B10] Elliott R. Bohart A. C. Watson J. C. Murphy D. (2018). Therapist empathy and client outcome: an updated meta-analysis. *Psychotherapy* 55 399–410. 10.1037/pst0000175 30335453

[B11] Freud S. (1900/1913). *The Interpretation of Dreams.* New York, NY: Macmillan.

[B12] Garfinkel H. (1967). *Studies in Ethnomethodology.* Englewood Cliffs, NJ: Prentice-Hall.

[B13] Gelso C. Carter J. (1985). The relationship in counseling and psychotherapy. *Counsel. Psychol.* 13 155–243. 10.1177/0011000085132001

[B14] Goffman E. (1959). *The Presentation of Self in Everyday Life.* London: Penguin.

[B15] Goffman E. (1966). *Behavior in Public Places: Notes on the Social Organization of Gatherings.* New York, NY: The Free Press.

[B16] Graf E.-M. Scarvaglieri C. Spranz-Fogasy T. (eds) (2019). *Pragmatik der Veränderung: Problem- und Lösungsorientierte Kommunikation in Helfenden Berufen.* Tübingen: Narr.

[B17] Hausendorf H. Quasthoff U. (2005). *Sprachentwicklung und Interaktion: Eine Linguistische Studie zum Erwerb von Diskursfähigkeiten.* Radolfzell: Verlag für Gesprächsforschung.

[B18] Heritage J. (1998). Oh-prefaced responses to inquiry. *Lang. Soc.* 27 291–334. 10.1017/S0047404500019990

[B19] Heritage J. (2013). “Epistemics in conversation,” in *The Handbook of Conversation Analysis*, eds Sidnell J. Stivers T. (Oxford: Wiley-Blackwell), 370–394. 10.1002/9781118325001.ch18

[B20] Heritage J. Atkinson M. (1984). “Introduction,” in *Structures of Social Action: Studies in Conversation Analysis*, eds Atkinson M. Heritage J. (Cambridge: Cambridge University Press), 1–15. 10.1093/actrade/9780198836421.003.0001

[B21] Heritage J. Raymond G. (2005). The terms of agreement: indexing epistemic authority and subordination in talk-in-interaction. *Soc. Psychol. Q.* 68 15–38. 10.1177/019027250506800103

[B22] Horvath A. Bedi R. (2002). “The alliance,” in *Psychotherapy Relationships that Work: Therapist Contributions and Responsiveness to Patients*, ed. Norcross J. C. (New York, NY: Oxford University Press), 37–69.

[B23] Jefferson G. (2004). “Glossary of transcript symbols with an introduction,” in *Conversation Analysis: Studies from the First Generation*, ed. Lerner G. H. (Amsterdam: Benjamins), 13–31. 10.1075/pbns.125.02jef

[B24] Koerfer A. Reimer T. Albus C. (2018). “Beziehung Aufbauen,” in *Kommunikative Kompetenz in der Medizin: Ein Lehrbuch zur Theorie, Didaktik, Praxis und Evaluation der Ärztlichen Gesprächsführung*, eds Koerfer A. Albus C. (Mannheim: Verlag für Gesprächsforschung), 814–851.

[B25] Labov W. Fanshel D. (1977). *Therapeutic Discourse: Psychotherapy as Conservation.* New York: Academic Press.

[B26] Lakoff R. T. (1982). “The rationale of psychotherapeutic discourse,” in *Handbook of Interpersonal Psychotherapy*, eds Anchin J. C. Kiesler D. (New York: Pergamon Press), 132–146.

[B27] Lee S.-H. Tanaka H. (2016). Affiliation and alignment in responding actions. *J. Pragmat.* 100 1–7. 10.1016/j.pragma.2016.05.008

[B28] Linke A. Schröter J. (2017). “Sprache in Beziehungen – Beziehungen in Sprache: Überlegungen zur Konstitution eines linguistischen Forschungsfeldes,” in *Sprache und Beziehung*, eds Linke A. Schröter J. (Berlin: de Gruyter), 1–32. 10.1515/9783110496918-002

[B29] Locher M. Watts R. (2008). “Relational work and impoliteness: negotiating norms of linguistic behaviour,” in *Impoliteness in Language*, eds Locher M. Bousfield D. (Berlin: de Gruyter), 77–100.

[B30] Mondada L. (2007). Multimodal resources for turn-taking: pointing and the emergence of possible next speakers. *Discourse Stud.* 9 195–226.

[B31] Muntigl P. (2020). Managing distress over time in psychotherapy: guiding the client in and through intense emotional work. *Front. Psychol.* 10:3052. 10.3389/fpsyg.2019.03052 32140117 PMC7042173

[B32] Muntigl P. Horvath A. (2014). The therapeutic relationship in action: how therapists and clients co-manage relational disaffiliation. *Psychother. Res.* 24 327–345. 10.1080/10503307.2013.807525 24716569

[B33] Muntigl P. Knight N. Horvath A. Watkins A. (2012). Client attitudinal stance and therapist-client affiliation: a view from grammar and social interaction. *Res. Psychother.* 15 117–130. 10.4081/ripppo.2012.119

[B34] Muntigl P. Knight N. Watkins A. Horvath A. Angus L. (2013). Active retreating: person-centered practices to repair disaffiliation in therapy. *J. Pragmat.* 53 1–20. 10.1016/j.pragma.2013.03.019

[B35] Norcross J. C. Lambert M. J. (eds) (2018a). Evidenced-based psychotherapy relationships III. Special issue. *Psychotherapy* 55 303–315. 10.1037/pst0000193 30335448

[B36] Norcross J. C. Lambert M. J. (eds) (2018b). Psychotherapy relationships that work III. *Psychotherapy* 55 303–315. 10.1037/pst0000193 30335448

[B37] Norrick N. R. (2010). *Conversational Narrative: Storytelling in Everyday Talk.* Amsterdam: Benjamins.

[B38] Pawelczyk J. (2019). “Client change in psychotherapy: methodological challenges and analytical affordances of discourse analysis,” in *Pragmatik der Veränderung: Problem- und Lösungsorientierte Kommunikation in helfenden Berufen*, eds Graf E.-M. Scarvaglieri C. Spranz-Fogasy T. (Tübingen: Narr), 65–88. 10.1007/978-1-349-13825-8_5

[B39] Peräkylä A. (2004). Making links in psychoanalytic interpretations: a conversation analytic perspective. *Psychother. Res.* 14 289–307. 10.1093/ptr/kph026

[B40] Peräkylä A. (2005). Patients’ responses to interpretations: a dialogue between conversation analysis and psychoanalytic theory. *Commun. Med.* 2 163–176. 10.1515/come.2005.2.2.163 16808721

[B41] Peräkylä A. (2019). Conversation analysis and psychotherapy: identifying transformative sequences. *Res. Lang. Soc. Interact.* 52 257–280. 10.1080/08351813.2019.1631044

[B42] Redder A. (2008). “Functional pragmatics,” in *Handbook of Interpersonal Communication*, eds Antos G. Ventola E. (Berlin: Mouton de Gruyter), 133–178.

[B43] Rehbein J. Schmidt T. Meyer B. Watzke F. Herkenrath A. (2004). *Handbuch für das Computergestützte Transkribieren nach HIAT.* Hamburg: Sonderforschungsbereich, 538.

[B44] Ribeiro E. Ribeiro A. P. Goncalves M. M. Horvath A. Stiles W. B. (2013). How collaboration in therapy becomes therapeutic: the therapeutic collaboration coding system. *Psychol. Psychother.* 86 294–314. 10.1111/j.2044-8341.2012.02066.x 23955792

[B45] Rogers C. R. Lyon H. C. Tausch R. (2013). *On Becoming an Effective Teacher: Person-Centred Teaching, Psychology, Philosophy, and Dialogues with Carl R. Rogers.* Abingdon: Routledge.

[B46] Sacks H. (1995). *Lectures on Conversation*, Vol. I & II Oxford: Blackwell.

[B47] Sacks H. Schegloff E. A. (1979). “Two preferences in the organization of reference to persons in conversation and their interaction,” in *Everyday Language: Studies in Ethnomethodology*, ed. Psathas G. (New York, NY: Irvington), 15–21.

[B48] Scarvaglieri C. (2013a). *Nichts Anderes als ein Austausch von Worten: Sprachliches Handeln in der Psychotherapie.* Berlin: de Gruyter.

[B49] Scarvaglieri C. (2013b). “Sprachliches Veranschaulichen und kuratives Verstehen in der Psychotherapie,” in *Veranschaulichungsverfahren im Gespräch*, eds Birkner K. Ehmer O. (Mannheim: Verlag für Gesprächsforschung), 66–92.

[B50] Scarvaglieri C. (2017). “Beraten und Psychotherapie: Zur Differenzierung zweier Formate helfenden Handelns,” in *Beraten in Interaktion: Eine gesprächslinguistische Typologie des Beratens*, ed. Pick I. (Frankfurt: Lang), 53–76.

[B51] Scarvaglieri C. (2019a). Starting points for therapeutic change: therapists’ rewordings of patients’ experiences. *Commun. Med.* 16:2. 10.1558/cam.33827 39916021

[B52] Scarvaglieri C. (2019b). “Veränderung durch Verstehen in der Psychotherapie,” in *Pragmatik der Veränderung: Problem- und Lösungsorientierte Kommunikation in helfenden Berufen*, eds Graf E.-M. Scarvaglieri C. Spranz-Fogasy T. (Tübingen: Narr), 121–145.

[B53] Schiffrin D. (2006). *In Other Words: Variation in Reference and Narrative.* Cambridge: Cambridge University Press.

[B54] Sidnell J. Stivers T. (eds) (2013). *The Handbook of Conversation Analysis.* Oxford: Wiley-Blackwell.

[B55] Stivers T. (2008). Stance, alignment, and affiliation during storytelling: when nodding is a token of affiliation. *Res. Lang. Soc. Interact.* 41 31–57. 10.1080/08351810701691123

[B56] Stivers T. Mondada L. Steensig J. (2011). “Knowledge, morality and affiliation in social interaction,” in *The Morality of Knowledge in Conversation*, eds Steensig J. Mondada L. Stivers T. (Cambridge: Cambridge University Press), 3–24. 10.1017/cbo9780511921674.002

[B57] Storck T. Buchholz M. Lindner R. Kächele H. (2020). Perspektiven der psychodynamischen Prozessforschung. *Forum der Psychoanalyse* 36 71–85. 10.1007/s00451-020-00382-w

[B58] Tannen D. Ehernberger Hamilton H. Schiffrin D. eds (2015). *The Handbook of Discourse Analysis*, 2 Edn Hoboken, NJ: Wiley Blackwell.

[B59] Tay D. (2013). *Metaphor in Psychotherapy: A Descriptive and Prescriptive Analysis.* Amsterdam: Benjamins.

[B60] Turner R. (1972). “Some formal properties of therapy talk,” in *Studies in Social Interaction*, ed. Sudnow D. (New York: Free Press), 367–396.

[B61] Vehviläinen S. (2003). Preparing and delivering interpretations in psychoanalytic interaction. *Text* 23 573–606.

[B62] Volosinov V. (1930/1973). *Marxism and the Philosophy of Language.* New York: Seminar Press.

[B63] Voutilainen L. Federico R. Peräkylä A. (2018). “Conversation analysis and psychotherapeutic change,” in *Longitudinal Studies on the Organization of Social Interaction*, eds Pekarek Doehler S. Wagner J. González-Martínez E. (London: Palgrave Macmillan), 225–254. 10.1057/978-1-137-57007-9_8

[B64] Weingarten R. (1990). “Reformulierungen in der Gesprächspsychotherapie,” in *Medizinische und Therapeutische Kommunikation: Diskursanalytische Untersuchungen*, eds Ehlich K. Koerfer A. Redder A. Weingarten R. (Opladen: Westdeutscher Verlag), 228–240. 10.1007/978-3-663-14675-9_16

[B65] Weiste E. Voutilainen L. Peräkylä A. (2016). Epistemic asymmetries in psychotherapy interaction: therapists’ practices for displaying access to clients’ inner experiences. *Sociol. Health Illness* 38 645–661. 10.1111/1467-9566.12384 26574238

[B66] Yamaguchi M. (2005). Discursive representation and enactment of national identities: the case of generation 1.5 Japanese. *Discourse Soc.* 16 269–299. 10.1177/0957926505049624

